# A qualitative content analysis exploring the portrayal of antibiotic use and antibiotic resistance in UK smallholding print media

**DOI:** 10.3389/fvets.2025.1570090

**Published:** 2025-07-30

**Authors:** Claire D.A. Scott, Irene Bueno, Alex J. Tasker, Henry Buller, Kristen K. Reyher

**Affiliations:** ^1^The Bristol Veterinary School, University of Bristol, Langford, United Kingdom; ^2^Department of Geography, University of Exeter, Exeter, United Kingdom

**Keywords:** antibiotic use, antibiotic stewardship, content analysis, smallholder, thematic analysis

## Abstract

**Background:**

Antibiotic use (ABU) practices and attitudes around antibiotic resistance (ABR) are relatively unstudied for smallholders in the UK. Due to differences in outlook, goals and farming methods, these factors may differ from commercial farmers. To gain insight into how the issues of ABU and ABR are communicated amongst and for smallholders, we completed a qualitative content analysis of smallholding print media.

**Methods:**

To explore how the concepts of ABR and ABU were portrayed, we gathered 129 articles from four UK smallholding magazines published from January 2015 to December 2019; material relating to ABR (from all issues) and ABU (from quarterly issues) was extracted. Guided by framing theory, we identified key themes and sub-themes. We then used qualitative relational content analysis to consider how and when themes and sub-themes appeared together.

**Results:**

In the theme ‘Antibiotic stewardship’, contributors encouraged practices such as seeking veterinary supervision for ABU or preventing the need for antibiotics for farm animals. In the theme ‘Antibiotics for livestock health’, contributors described the importance of antibiotics to protect animal welfare. ‘Antibiotic stewardship’ occurred alongside ‘Antibiotics for livestock health’ two-thirds of the time, meaning that reference to antibiotic stewardship was common when discussing ABU. Whilst ABU on smallholdings was characterised as infrequent and broadly restricted to singular animals after observation of clinical signs of disease, analysis of reported instances of ABU showed that recommendations described in the theme ‘Antibiotic stewardship’ may not consistently be completed in practice, including seeking veterinary supervision for ABU. In the theme ‘Problems are elsewhere’, contributors ascribed greater significance to groups such as commercial farming or human medicine in their overuse of antibiotics and hence contribution to ABR. Especially where the ‘Problems are elsewhere’ theme occurred alongside ‘Antibiotic stewardship’, contributors appeared to demonstrate a lack of acceptance of responsibility for ABR which ranged from subtle to more overt.

**Conclusion:**

Our study provides insight into the ways smallholders consider, discuss and use antibiotics in the context of and in relation to ABR. We identify potential facilitators and barriers to antibiotic stewardship on smallholdings and suggest recommendations for how educational material aimed at smallholders could be adapted to better encourage antibiotic stewardship practices.

## Introduction

In response to growing public awareness and pressure around the global health threat of antibiotic resistance (ABR) (1, 2), those acting within and alongside the UK commercial farming sector have mounted concerted efforts to reduce and refine antibiotic use (ABU) (3–5). As a result, by 2023, UK sales of antibiotics for use in food-producing animals had decreased by 59% [adjusted for animal population) since 2014 (Veterinary Medicines (6)]. ABU on UK smallholdings is, however, relatively unstudied in comparison to ABU on commercial farms (7–12).

Although globally accepted or appropriate definitions for the terms ‘smallholding’ or ‘smallholder’ do not exist (13), some authors have outlined what it means to be a smallholder in the UK. Authors discuss smallholding as marginal or ‘alternative’ to commercial farming, often with goals of self-sufficiency, generally not providing a sole income and likely keeping small numbers of animals of several different species (14–17).

The suggested differences between smallholdings and commercial farms in terms of outlook, goals and farming methods (15) means that practices and trends around ABU as well as attitudes around ABR may differ. Hibbard et al. (18) define antibiotic stewardship as using and prescribing antimicrobials in humans and animals in a way that ensures the availability of antimicrobials for individuals now and in the future. Given differences between smallholdings and commercial farms, we contend that antibiotic stewardship recommendations may need to be contextualised for use in smallholdings; this may include the development and deployment of alternative approaches.

Without pressures of intensification and often adopting minimal input, organic and ‘chemical-free’ practices (15, 16), smallholder systems may differ from the more antibiotic-permissive practices which have, traditionally, been associated with the UK commercial farming sector (19–22). UK smallholders have been shown to believe that their practices – such as close animal supervision – allowed them to decrease disease risk (23), thereby reducing their need for antibiotics (12). Further, some veterinarians providing services to UK smallholders considered that smallholdings posed a ‘lower risk’ of inappropriate ABU due to their extensive systems of animal management (generally housing animals outdoors at low stocking densities), which they believed resulted in lower disease occurrence and antibiotic need (24). Veterinarians also regarded that more instances of ABU on smallholdings were likely to be supervised by a veterinarian than on commercial farms (24).

That being said, despite also clearly articulating their antibiotic stewardship goals and intentions, smallholders have been shown to characterise ABU on smallholdings as part of a first-line response to non-specific clinical signs of disease or to prevent future infections (12). Reasons smallholders gave to justify more precautionary ABU included difficulties diagnosing the bacterial cause of clinical signs of disease as well as the speed at which animals could deteriorate in condition if not promptly treated with antibiotics. Further, veterinarians providing services to smallholders have described feeling limited in the extent to which inappropriate ABU is preventable on smallholdings, due to challenges diagnosing the cause of clinical signs of farm animals as well as difficulties ensuring that all instances of ABU are supervised by a veterinarian (24). In the UK, antibiotics may only be administered to farm animals under the supervision of a veterinarian, following a clinical assessment of the animal or group of animals to which they will be administered (25). Veterinarians are permitted to prescribe antibiotics that can be left on farms and used in stipulated ways (25, 26) by suitably trained stockpersons (7, 27, 28). Research has, however, identified that some smallholders and commercial farmers use antibiotics without veterinary supervision (11, 12).

Given that fewer studies have examined ABU on UK smallholdings in comparison to commercial farms, we sought to gain further insight into the opinions, attitudes and practices communicated by, to and for UK smallholders regarding the issues of ABR and ABU. We chose to take inspiration from studies over the last 20 years which analysed secondary sources to provide insights into ABR and ABU issues (15, 29–33). With these prior studies in mind, we utilised the method of a print media-focused content analysis (34) to understand how the issues of ABR and ABU were constructed, framed and presented in smallholder print media over five years. Our study had three aims. Firstly, to understand how ABU and ABR were conceptualised and operationalised for smallholders, including how contributors framed the issue of agency for ABR (29). Secondly, taking inspiration from Doidge et al. (30), we aimed to develop recommendations for those producing future educational material aimed at smallholders to support antibiotic stewardship goals. Finally, taking inspiration from Holloway (15), we aimed to offer between-method triangulation of facilitators and barriers of antibiotic stewardship and antibiotic stewardship recommendations identified in our related research examining UK smallholdings (11, 24, 32).

## Methods

### Selection of analytical materials

The existence of varied publications aimed specifically at UK smallholders suggested that print media was a source of relevant data to address our core research questions. Although analysis of posts within smallholding social media groups could have provided another, rich form of data (as was achieved by (27) who analysed a public social media post involving commercial farmers), the ‘private’ nature of these smallholding groups means that analysis of such posts would have contravened recognised ethics guidelines for internet-mediated research (35).

We selected print media publications to reflect both generalist and specialist audiences at a national scale: ‘Country Smallholding’, ‘The Smallholder’, ‘Practical Poultry’ and ‘Practical Pigs’. Two of the selected publications – ‘Country Smallholding’ and ‘The Smallholder’ – had targeted audiences of the entire UK smallholding community. Earlier editions had also been selected for analysis in smallholding research by Holloway (15). ‘Country Smallholding’ described itself as ‘Britain’s best-selling smallholding magazine’ and was published monthly throughout our reading frame (36). ‘The Smallholder’ was referred to as ‘the original smallholding magazine’ (37). It was launched in 1910 and was published bimonthly with special issues. ‘Country Smallholding’ and ‘The Smallholder’ merged in September 2022, after the reading frame for this study (36). Two further publications had more specific target audiences, reflective of key species groups within smallholding. ‘Practical Poultry’ was referred to as the ‘UK’s best-selling chicken magazine’ (38) whilst ‘Practical Pigs’ was described as ‘the definitive guide to keeping and rearing pigs’ (39). ‘Practical Poultry’ was published monthly until mid-2018, at which point it moved to one issue every 2 months. ‘Practical Pigs’ was published seasonally. All four publications regularly included expert smallholder features; accounts of first-hand smallholding experience; news articles on current smallholding topics; articles from veterinarians; and questions put by smallholders to the magazines’ resident veterinarians or smallholding experts.

### Selection of analytical methods

Framing is a method employed to analyse how issues and events are portrayed by contributors to media forms (40, 41). To complete frame analysis, researchers consider how contributors selected and promoted specific information or acted to convince readers of a particular interpretation or perspective on an issue, which may provide insights into cultural values and shared narratives within a community (42). In the version developed by Benford and Snow (43), frame analysis involves exploring the three framing tasks of collective action: diagnostic framing – identification of a problem, its cause and attribution of blame; prognostic framing – articulation of solutions and the strategies to achieve such solutions; and motivational framing – rationale or impetus for action.

Providing inspiration for the current study, Morris et al. (32) explored how relationships between agricultural ABU and problems posed by ABR were constructed into three dominant ‘frames’ across four examples of UK print media, including one from the farming press. As a result of their analysis using Snow and Benford’s technique, Morris et al. (32) described how the frames they identified represented opposing views regarding the implications of ABU in agriculture on human ABR.

Although our aim was to understand how the issues of ABR and ABU were framed in smallholder print media over 5 years, the articles extracted for analysis during this study were broad in character meaning that they were not consistently amenable to frame analysis, as some contributors did not appear to be attempting to persuade readers to a certain belief. Rather than this detracting from our methodology, the inclusion of such broad material enabled exploration of how frames translated into accounts of actual ABU practices. Therefore, instead of using a framing approach alone, we took inspiration from Dreser et al. (31) and used frame analysis to guide our content analysis. We also took inspiration from these authors’ use of quantitative content analysis to understand the frequency at which topics arose and the frequency of different stakeholder voices. We used a qualitative relational content analysis to consider the relationships between concepts identified in our study (34, 44). As a framework for our approach, we completed our research in line with the READ approach for document analysis (45).

### Data extraction

Five years of back issues from January 2015 to December 2019 were examined for each journal, by purchasing back issues of each publication. The start date of January 2015 was chosen to coincide, first, with the start of a major expansion of awareness and interest in ABU in UK livestock farming [as demonstrated by the establishment and initial publications of the Committee on Antimicrobial Resistance, known as the O’Neill Committee (1)] and, secondly, with the beginning of a concerted period of decreasing ABU in the major UK livestock sectors (46). The end date of December 2019 was chosen for reasons of resource constraints.

As per step one of the READ approach for document analysis (45), the lead researcher (CS) skim-read all publications manually (due to the lack of a search term function for the online versions of publications). This was to identify and extract any content relating to ABR (whether applicable to commercial livestock agriculture or smallholding) from all issues. Owing to the much more frequent appearance of discussions around ABU in comparison to ABR, we extracted content relating to ABU from all quarterly issues of the publications, including advertising text and references to trade names or ingredients of antibiotics. Therefore, for seasonally issued magazines, all issues were read for extraction of content related to ABR and ABU. For the monthly and bi-monthly magazines, January, April, July and October issues were read to extract content related to ABR and ABU; all other issues were read for extraction of content just related to ABR. In all, 129 distinct articles were identified which were considered relevant to discussions of ABU and ABR.

### Data analysis

Next, we completed a content analysis, including both qualitative and relational elements (34, 44). According to steps two and three of the READ approach for document analysis (45), all articles were uploaded to NVivo (47). After re-reading the extracted data to build familiarity, the first author (CS) performed inductive coding within NVivo to produce 71 individual codes. This was a subjective process in which CS generated and applied analytical descriptions (codes) to segments of data which she felt were relevant or meaningful to the issues of ABU and ABR on smallholdings. Whilst deriving codes, CS considered the three framing tasks of collective action, in particular: the problem being described and where blame was being attributed; the solutions that were being encouraged; and why contributors were encouraging such action.

CS reduced the number of codes to 51, by merging codes she considered similar. As an example, the code ‘antibiotics have improved animal health’ was merged with ‘antibiotics are important for animal health’. After re-reading the data to ensure that the reduced number of codes were representative of the data they were ascribed to, codes were organised into three main themes and further sub-themes. This was achieved by grouping similar codes as to their perceived intention (again considering the three framing tasks) or subject area and grouping codes which often appeared together. Multiple code and theme configurations were trialled by CS, until a configuration was arrived at which she felt best summarised the concepts relating to ABU and ABR described in the data and articulated patterns of shared meaning. Throughout this process, CS regularly considered how her positionality may have affected her analytical decisions and considered alternative layers of meaning. This was also frequently prompted by her supervisors and co-authors of this paper. A positionality statement for CS can be found on page 83 of CS’s doctoral thesis (48) and a shortened version is provided in the next section.

**Figure 1:**
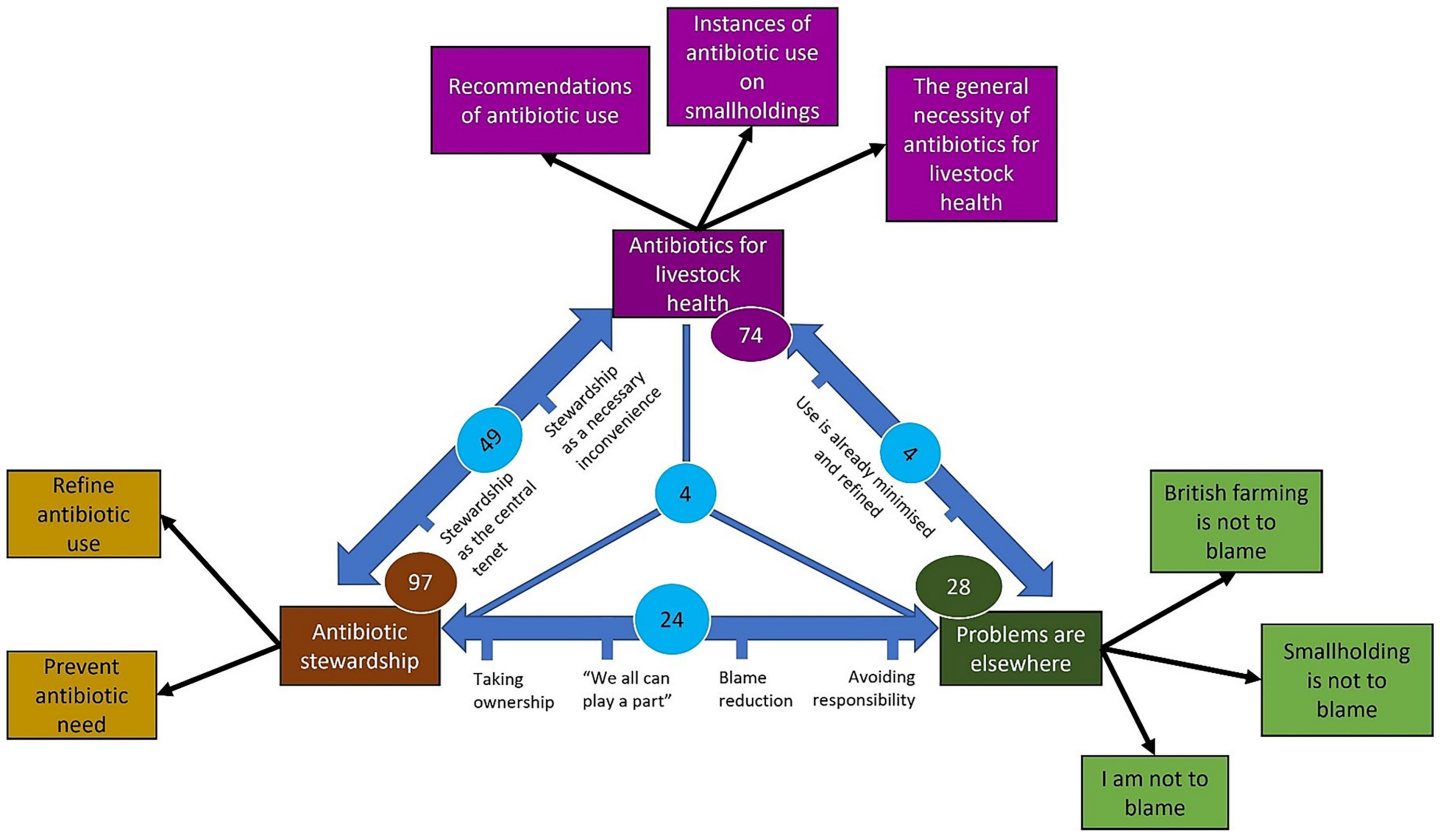
Graphical representation of each theme, the sub-themes relating to each theme, the frequency of each theme and the relationships between themes. The number of times each theme occurred is represented in coloured circles. The number of times themes appeared together is shown in blue circles. The reasons for themes appearing together and the spectra along which articles appeared between two themes are shown alongside the blue arrows.

Following theme generation, data were typed into Microsoft Excel (49), re-read and articles were assigned to (often multiple) themes. To explore numerical relationships between themes and whether these changed over time, we examined the typed data within Microsoft Excel. We derived descriptive statistics detailing when themes, or multiple themes, occurred; these were stratified for each magazine, species, year and contributor type. Finally, themes, codes and the relationship between them were refined and formulated into the graphical representation shown in Figure 1, as per step four of the READ approach (45) and as is common for relational content analyses (34, 44).

### Positionality statement

CS is a white, British female who grew up in a suburban locality. After qualifying as a veterinarian, she worked for 2 years as a pig veterinarian, where she worked with both commercial and smallholding pigs and their keepers. Observing both different and similar challenges and opportunities for health and antibiotic stewardship on these holdings led her to constantly compare and contrast the many systems of pig keeping present in the UK.

Her interest in ABR and ABU stemmed from a school research project which enabled discussion of these issues with farmers, exploring their experiences of ABU and perceptions of antibiotic need on their farms. Animal ABR became her subject of interest which she periodically revisited up to starting her PhD project: exploring ABU and ABR on smallholdings in the UK.

## Results and discussion

The number of articles extracted from each source magazine, for each species, for each year, from each contributor type and for each theme and theme combination is shown in Table 1. This table allows appraisal of how themes occurred together, under what conditions themes appeared and details trends for each theme over time. Some elements of this table, however, should be compared with caution. For example, we extracted articles for mention of ABR from every issue, but for ABU only from quarterly issues. If every issue had been extracted for articles related to both ABU and ABR, it is likely that the theme ‘Antibiotics for livestock health’ would have appeared relatively more often, given that other themes were more relevant to discussions around ABR. The relative number of occurrences of themes, therefore, should be compared carefully. The relative number of articles extracted per magazine should also be compared carefully, due to the more regular publication of some magazines relative to others and the different lengths of publications. The number of pig- and poultry-specific articles was reflective of the inclusion of two publications targeting these audiences specifically. Some articles had more than one contributor type and, therefore, the total of articles described under contributor types is more than the total number of articles extracted. The highest number of articles extracted across all themes occurred in 2016, reflecting the emergence of a focus on the issues of ABR and ABU in the wider press and policy debate at that time.

**Table 1:** Number of articles extracted from each source magazine for each species and year, from each contributor type and for each theme and theme combination.

	Antibiotic stewardship			Antibiotics for livestock health		Problems are elsewhere	Outliers	Total
	Total	With antibiotics for livestock health	With problems are elsewhere	Total	With problems are elsewhere	Total		
Magazine								
Country Smallholding	32	24	8	35	4	9	2	46
Practical Pigs	31	12	7	16	0	8	0	36
Practical Poultry	21	7	7	13	0	9	1	29
The Smallholder	13	6	2	10	0	2	1	18
Species								
Camelid	2	0	0	1	0	0	0	3
Cattle	0	0	0	1	0	0	0	1
General species	16	7	6	8	4	8	0	19
Pig	34	15	7	20	0	8	0	40
Poultry	37	21	8	33	0	4	4	53
Sheep and goats	8	6	3	11	0	2	0	13
Contributor type								
Advertisement	6	0	0	0	0	0	0	6
Industry representative	9	2	5	2	1	6	0	10
Journalist	15	3	6	4	1	7	0	17
Layperson	10	9	1	9	0	1	1	11
Smallholder writer	38	22	11	39	2	13	3	59
Veterinarian	28	22	3	30	2	4	0	37
Year								
2015	17	11	4	18	1	5	2	26
2016	35	14	14	19	2	15	1	42
2017	15	11	4	13	1	6	0	19
2018	13	6	0	11	0	0	0	18
2019	17	7	2	13	0	2	1	24
Total	97	49	24	74	4	28	4	129

Using the methods described, we derived three themes and further sub-themes to which articles broadly conferred. Four articles were outliers, meaning that they did not confer any of the themes. For example, two articles focused on anthelmintic resistance and mentioned ABR only to provide a metaphor. Figure 1 is a graphical representation of the sub-themes under each theme, the frequency of each theme and the relationships between themes.

### Theme 1: antibiotic stewardship

In Theme 1, contributors described goals, recommendations and practices which they regarded as beneficial to achieving ‘Antibiotic stewardship’ in livestock, as is demonstrated by the following quote:

*“We all have a responsibility to be proactive in reducing usage on our farms/smallholdings where possible;* […] *to achieve this we should aim for improved management and the use of vaccines where appropriate; and* […] *where we do have to give antibiotics, animals should receive the most appropriate antibiotic for the disease being treated, at the correct dose and for the correct amount of time.”* (Smallholder Writer, Country Smallholding, September 2017)

This theme was commonly expressed by veterinarians, smallholders, industry bodies such as the Soil Association (an organic farming body in the UK) and through advertising material. Strategies to achieve antibiotic stewardship broadly fell into two categories.

#### Sub-theme 1: prevent antibiotic need

For this sub-theme, contributors recommended that *“prevention is better than cure”* (Smallholder Writer, Country Smallholding, Spring 2017) in terms of appropriate ABU. Smallholder systems were described as holding the tools by which prevention could be achieved, utilising ‘natural’ farming methods alongside close animal supervision – both goals that have been reflected in other work examining UK smallholders (12, 15, 23). Contributors characterised this ‘natural-ness’ as keeping animals in outdoor, non-intensive systems – thereby protecting animals against stress – and using natural products to improve health.

*“By thinking about the welfare of the animal, giving it a more natural life, it has the best chance of fighting off infection by itself.”* (Industry Representative, Country Smallholding, November 2016)

Whether smallholders were correct in their assertion that these practices may reduce infectious diseases and the need for antibiotic treatment is unknown, however smallholders and veterinarians characterised UK smallholding ABU as likely to be lower than on commercial farms in related work (12, 24). Further, in answer to a survey of backyard poultry keeper attitudes toward poultry health and biosecurity, 60.2% of keepers reported that they had never used antibiotics on their birds (*n* = 145) (50), suggesting that ABU may be low. In agreement, extensive systems (involving keeping animals outdoors at low stocking densities, which is commonly practiced on smallholdings) have been linked to lower ABU within commercial farming (51, 52).

That being said, whether these approaches contribute to low ABU on smallholdings may also depend on the extent to which ‘natural-ness’ is practised. Although vaccination of animals was described by many contributors as a mechanism to ‘Prevent antibiotic need’, other contributors suggested that ‘natural-ness’ may include the rejection of vaccinations.

*“Among those who find the hybrid world a somewhat sterile place, there will be those who question the long-term effects on natural immunity of vaccinating generation after generation of potential breeding stock.”* (Smallholder Writer, Practical Poultry, October 2016)

Uptake of vaccination amongst some UK smallholding groups has been documented as variable (12, 50, 53–55). In a related work, smallholders cited wanting to provide animals with a more natural existence as one reason to not vaccinate their farm animals (12).

Vaccination has been presented in international guidance as a key measure to achieving antibiotic stewardship (56). This sentiment has been reflected in farming literature (57) and is a view that has been described amongst pig farmers and pig veterinarians (58, 59). That being said, Davies et al. (51) found no significant correlation between abortion vaccine use or use of a footrot vaccination and ABU for a population of UK sheep flocks. Given this, it is important to keep in mind that administering vaccinations on smallholdings may not always lead to improved antibiotic stewardship.

#### Sub-theme 2: refine ABU

Under this sub-theme, contributors recommended that antibiotics should be used only where absolutely necessary and with appropriate administration. Contributors also frequently informed readers of the need to ensure that ABU was under the direction of a veterinarian, who were described as the guardians of appropriate ABU.

*“If your vet prescribes a course of antibiotics, always finish the course.* […] *Never store up spare antibiotics for a rainy day. Always read the label or prescription.* […] *Only use medicines that are in date and have been stored correctly.”* (Veterinarian, Country Smallholding, September 2015)

The extent that these recommendations appeared to be practiced during the instances of ABU which contributors described is explored in a later theme of this manuscript.

#### Theme 1 motivations

The most frequent motivation discussed by contributors recommending ‘Antibiotic stewardship’ was a goal to reduce the presence of ABR-bacteria in both humans and animals, in line with public health concerns.

*“It’s been called an antibiotic apocalypse with at least 50,000 people dying each year in Europe and the US alone from infections that antibiotics have lost the power to treat. Bacteria are developing resistance at such an alarming rate it has been estimated that antibiotics may only remain effective for just the next few decades.”* (Smallholder Writer, Country Smallholding, November 2016)

Rather than abdicating responsibility for ABR – as has been described elsewhere in the UK national and farming press (29, 32) – many contributors under this theme accepted a level of responsibility for human ABR, in terms of their own antibiotic stewardship or the encouragement of antibiotic stewardship among others. Unlike we describe in a later theme – where smallholders detailed a lesser responsibility than commercial farmers – many contributors under this theme conveyed shared responsibility for the issue across the farming sector.

*“All our pigs are as much of the national herd as those owned by the commercial guys.”* (Smallholder Writer, Practical Pigs, Winter 2016)

Some contributors also provided alternative motivators for reducing ABU such as cost savings, protection of animal welfare and provision of food reared using ‘natural’ methods. For example, cost reduction was commonly cited as a reason to employ benchmarking for ABU via the newly introduced (2016) electronic medicine book for pigs, as this would enable keepers to critically examine each instance of ABU over the previous time period (60).

*“Once you begin to input data, the system generates reports and trends displayed in graph formats. Most of us think we use antibiotics as the last option to treat a sick animal, but monitoring the trends may well bring another layer of cost-saving prevention.”* (Smallholder Writer, Practical Pigs, Winter 2016)

Farmers have been described as regarding the achievement of reductions in the cost of medicines a strong contributor to their actions around reducing ABU (61). As described by Morris et al. (32), the provision of alternative motivators may more successfully achieve voluntary action amongst readers in terms of improving antibiotic stewardship, especially amongst readers who aren’t convinced of the significance of livestock ABU for human ABR.

### Theme 2: antibiotics for livestock health

This theme, commonly expressed by veterinarians and smallholders, highlighted the importance of antibiotics within smallholding and farming communities by describing a critical need for ABU for the protection of livestock health [see also (58, 59)].

*“For lambing time there’s a whole list of ‘must haves’ ranging from penicillin-based antibiotic, propylene glycol* […]." (Smallholder Writer, Country Smallholding, February 2016)

Although contributors did not consistently describe their motivations for expressing this theme – this theme was generally less amenable to frame analysis than the previous theme – the impetus was generally implied to be to protect farm animals from disease, thereby conserving health and welfare. Under this theme, we identified three sub-themes.

#### Sub-theme 1: recommendations for ABU

Under this sub-theme, contributors detailed potential or clear recommendations of antibiotic need, in answer to smallholder questions or whilst describing disease conditions or clinical signs of disease which could be experienced by farm animals. This sub-theme appeared 47 times and, out of those, it appeared alongside Theme 1 (‘Antibiotic stewardship’) 25 times. This showed that recommendations for ABU were qualified with some consideration of antibiotic stewardship over half the times that this sub-theme appeared. The specific ‘Antibiotic stewardship’ technique contributors recommended alongside this sub-theme was most often that a veterinarian should be consulted before ABU. For example, when describing suitable treatment for a vulval injury in a pig, one contributor said:

*“This should involve isolation of the animal plus local antisepsis and the possible use of suitable antibiotic. Such carefully selected antibiotic may well vary from pig farm to pig farm and must be used under strict guidance from your vet.”* (Veterinarian, Practical Pigs, Autumn 2016)

Within the articles in which contributors encouraged veterinary involvement or other antibiotic stewardship recommendations, however, there was a spectrum, from those where antibiotic stewardship was characterised as a central tenet (as above), to those where antibiotic stewardship was described as a necessary inconvenience. On the latter end of the scale, for example:

*“Check your birds for any signs of winter colds/sneezing and try to obtain some Tylan soluble (a yellow powder antibiotic from your vet), which is a POM (prescription-only medicine). This means, unfortunately, that you will have to encounter the odd problem and pay for a vets appointment before being able to purchase any of this effective antibiotic.”* (Smallholder Writer, Country Smallholding, January 2015)

In terms of the conditions for which contributors recommended ABU, this was generally following diagnosis of the cause of the clinical signs of disease being observed, in terms of bacterial species.

*“Urgent veterinary attention is required for affected sheep* [with pasteurellosis] *and, in some cases, early antibiotic treatment can save these animals.”* (Veterinarian, Country Smallholding, October 2019)

Occasionally these conditions were viral rather than bacterial and, in these cases, contributors described ABU as sensible to protect animals from secondary bacterial infections [see also (59)].

In terms of the types of antibiotics recommended, reflective of findings in a related work (12), there was only one mention of a European Medicines Agency (EMA) Category B antibiotic, which should be ‘Restricted’ for use in animals to mitigate the risk to public health (62). In this case, this was a fluoroquinolone antibiotic:

“*Any discharge from the nostrils is symptomatic of a sinus infection; also puffy cheeks. Antibiotic from the vet, such as Baytril will be needed.”* (Smallholder Writer, The Smallholder, September 2017)

There were 16 mentions of EMA Category C antibiotics, for which ‘Caution’ should be applied and which should be used only when there are no Category D ‘Prudence’ antibiotics that would be clinically effective (62). The most common Category C ‘Caution’ antibiotic mentioned was tylosin – a macrolide antibiotic. This antibiotic was most often recommended for treatment of suspected mycoplasmal infection in birds; such an antibiotic has also been suggested as suitable for this condition in backyard poultry in the veterinary literature (63). Reflecting how non-clinical factors such as the withdrawal period can be an important influence on antibiotic choice (see also [59, 64)], a veterinarian wrote:

*“If you want to use antibiotics in a laying hen there are a few that are not only licensed but have nil egg withdrawal: Tylan soluble [tylosin] is one.”* (Veterinarian, Country Smallholding, October 2017)

UK commercial farmers are increasingly restricted by farm assurance scheme standards and supermarket contracts in terms of the antibiotics they are allowed to use, especially without laboratory evidence, and macrolides are being added to restricted lists (65). Veterinarians have described that their antibiotic prescribing practices are impacted by these restrictions in other farming research (64) and use of Highest-Priority Critically Important Antibiotics (HP-CIAs; antibiotics considered vital to human healthcare by the EMA) has reduced dramatically for farm animals in the UK following the introduction of more restrictive measures (6). However, findings in the current study may reflect research examining veterinarian prescribing for smallholder chickens in a companion animal veterinary environment in which 43.8% of the antibiotics prescribed included antibiotics considered HP-CIAs (66). As smallholders are less likely to be governed by farm assurance scheme standards, use of EMA Category B or C antibiotics may be higher (as a percentage to total antibiotics used) on smallholdings than on commercial farms.

#### Sub-theme 2: instances of ABU on smallholdings

Under this sub-theme, contributors described 16 instances of ABU which they reported to have taken place on smallholdings. Fifteen reports were described by smallholders and one was by a veterinarian. Articles took the form of reflective pieces or ‘ask the expert’ style questions, in which contributors asked veterinarians or experienced smallholders for advice on clinical cases. The instance of ABU described by a veterinarian detailed the use of a topical antibiotic spray on a smallholding. All other described instances of smallholding ABU were for injectable or oral administrations.

As was often recommended under Theme 1 (Antibiotic stewardship) and Theme 2, Sub-theme 1 (Recommendations for ABU), 11 of the 16 instances of ABU were characterised by contributors to have been under veterinary supervision. The other five instances of ABU appeared to have been completed without veterinary oversight. As discussed previously, veterinarians are permitted to leave antibiotics on farms in specific circumstances, however, these must only be used as prescribed by a veterinarian – for a specific animal (or a specific group of animals) for a specific condition (25, 26). Whilst frequent responsible medicines usage training is now a requirement for the UK’s leading farm assurance body, Red Tractor, and is a key target for the Responsible Use of Medicines in Agriculture Alliance (67), research has highlighted that antibiotics may be used outside of veterinary advice on both commercial farms and smallholdings (11, 12). In the current study, although contributors occasionally described some form of checks by veterinarians around smallholder competence to administer antibiotics before leaving antibiotics on-site, the nature of this was unclear.

*“We also keep injectable antibiotics and your vet will only prescribe it if they know you are able to use it appropriately.”* (Smallholder Writer, Country Smallholding, February 2016)

Guardabassi et al. (68) detail the logical thinking process (also known as a clinical reasoning approach) which veterinarians are required to complete in order to achieve antibiotic stewardship. Key to this is constructing and validating case definitions to arrive at most likely diagnoses and aid careful selection of a first-line antibiotic, if necessary. Under this sub-theme, contributors described ABU as targeted toward a particular diagnosis of bacterial disease in four out of 16 cases. In each of the four cases, this was for suspected mycoplasmal infections in birds; contributors did not report completion of diagnostic testing in any of these cases. In a further three cases, ABU appeared to be directed toward a clinical sign of disease rather than toward most likely bacterial causes, such as for keratitis in a goat:

“*At the first consultation the vet injected antibiotic into the lower lid causing it to swell and move the lashes away from the eye. A good result but would only be a temporary solution*.” (Smallholder Writer, The Smallholder, Summer 2016)

In further contrast to the recommendations described under Sub-theme 1 – that ABU should only take place following diagnosis of a specific condition requiring antibiotic treatment – in six out of the 16 instances, ABU was in response to non-specific clinical signs of disease, such as a high temperature.

*“The vet didn’t know what was causing it.* […] *She said she would blanket treat her – so she has been wormed, had anti-inflammatory treatment and antibiotics.”* (Lay Smallholder, Practical Poultry, January 2018)

Using antibiotics for non-specific clinical signs of disease, without prior establishment of the most likely diagnoses, may mean that antibiotics are prescribed when not indicated or that the most appropriate type of antibiotic is not selected (69). ABU based on non-specific clinical signs of disease has been commonly described on both UK smallholdings and commercial farms (24, 64, 70). Farm animal antibiotic prescribing has been documented to be influenced by clinical uncertainty and perceived pressure from farmers (10–12, 24, 59, 71, 72). Clinical uncertainty leading to antibiotic prescribing could be confounded for smallholdings by a lack of clinical guidelines aimed at smallholding animals (73) as well as general practice veterinarians often being required to supervise species outside of their normal areas of expertise (66).

In our study, three of the instances of ABU which were described to be aimed at very non-specific clinical signs of disease – a high temperature – also did not appear to be supervised by a veterinarian. These instances were portrayed in the same article as a quote from the smallholder writer’s veterinarian offering ‘Antibiotic stewardship’ advice:

“*Making a diagnosis is crucial in the decision-making process of whether antibiotics are appropriate.”* (Veterinarian, Country Smallholding, September 2017)

This example illustrates the discrepancies between the recommendations described within Theme 1 (Antibiotic stewardship) – to achieve a diagnosis prior to ABU – and the described instances of ABU when most likely diagnoses did not appear to be considered before ABU.

ABU instances described under the current sub-theme were, most often, reported for singular animals showing clinical signs of disease. However, we identified three instances of ABU which contributors described as aimed at preventing future infection. In a recent clarification (after the reading frame for this study), the UK Government’s Veterinary Medicines Directorate – responsible for the safety, quality and efficacy of veterinary medicines in the UK – stipulated that antibiotics must only be used for “prophylactic purposes in exceptional circumstances where the risk of an infection or of an infectious disease is very high and where the consequences of not prescribing the product are likely to be severe” and that they must not be used routinely or to compensate for suboptimal farm management (74, p. 1).

One of these instances of ABU aimed at preventing future infection was the only instance we found to describe topical ABU, detailed by a veterinarian; another was following surgery in a goat, which was administered under veterinary supervision. The final use of antibiotics aimed at preventing future infection we identified was the use of antibiotics as a routine, whole-herd measure to prevent bacterial infection in lambs.

*“We immediately give the lamb a squirt of Scour Halt. This is an oral anti-microbial which gives the lamb some protection from the bacteria in the bedding or on its mother’s udder, until its own immune system kicks in.”* (Smallholder Writer, The Smallholder, March 2017)

Although this particular product has since been discontinued for use in the UK (75), the routine administration of oral antibiotics as a preventative measure to newly born lambs has been analysed by Doidge et al. (30) after it was identified as a common practice amongst UK sheep farmers [see also (76)] despite contravening with best practice guidelines (74, 77).

In this particular description of the use of antibiotics as a routine, whole-herd measure to prevent bacterial infection in lambs, it is possible that the contributor did not understand that an ‘anti-microbial’ is an antibiotic. Examples of scientific misunderstanding were identified elsewhere in the publications, for example:

*“The same vet had visited him when he was just a young boar […]. He had contracted some kind of unidentifiable virus at the time and, after a painful shot of antibiotics, made a full recovery.”* (Smallholder Writer, Country Smallholding, April 2018)

It is also possible that more complex decisions were at play. Doidge et al. (30) discussed the emotional and nuanced decision-making process for farmers deciding whether to use antibiotics to prevent future infections; they identified that farmers’ intentions to use antibiotics in this way were sometimes reinforced by good intentions around animal welfare, to prevent animals from suffering from disease. Such findings were echoed by Coyne et al. (71) who reported that pig farmers and veterinarians supervising pig farms considered that the use of antibiotics to prevent conditions which cannot be controlled by other means was common, justifiable and prudent. Administration of antibiotics as a routine, whole-herd measure to prevent bacterial infection was not identified in related studies examining ABU on smallholdings (12, 24). This variation in findings may be reflective of factors such as a reading frame which commenced a decade ago in a quickly evolving field, or a different demographic of contributors to the articles analysed in the current study compared to smallholders and veterinarians surveyed in related studies.

Under the current sub-theme, contributors did not mention the type of antibiotic used in 10 of the 16 instances of ABU. Where mentioned, the EMA Category C antibiotic (‘Caution’) tylosin was the most frequently used antibiotic, which was discussed under Sub-theme 1 (Recommendations for ABU).

#### Sub-theme 3: the general necessity of antibiotics for livestock health

Within this sub-theme, contributors described antibiotics as critical to achieve good livestock health in general terms, in response to calls that ABU must be reduced. Contributors discussed how reductions in ABU must only be achieved *“while maintaining the health and welfare”* (Industry Representative, Practical Pigs, Summer 2018) of farm animals. Where this sub-theme arose alongside Theme 1 (‘Antibiotic stewardship’) – which was the case for nine out of the 10 times this sub-theme appeared – contributors portrayed the idea that, whilst antibiotic stewardship was important, antibiotic treatment for diseased animals was equally so.

*“There will inevitably be individual and group illnesses* […] *Veterinarians must retain the ability to prescribe proven pig antibiotics for diagnosed disease, where susceptible bacteria are known to be the cause or a major contributing factor.”* (Veterinarian, Practical Pigs, Spring 2016)

The idea of a ‘tipping point’ between reducing ABU and animal welfare problems has also been reflected in work examining UK commercial farming ABU. Doidge et al. (27) reported that some farmers felt unable to reduce ABU further without compromising animal welfare, as they had already reduced ABU to therapeutic use only. This sentiment is also echoed in findings by Jones et al. (61). In this way, the current sub-theme highlights how dilemmas encountered by those seeking to improve ABU in the commercial sector may also provide challenge to those with similar aims for smallholdings.

### Theme 3: problems are elsewhere

Within the theme ‘Problems are elsewhere’, contributors downplayed the significance of particular societal groups to ABU- and ABR-related concerns. The group from which contributors lessened blame varied from commercial farmers to smallholders themselves. The level of alleviation of blame within articles varied from subtle to more overt. As can be seen from Figure 1, articles appeared to lie along a spectrum from total ownership and shared responsibility for ‘Antibiotic stewardship’ to almost total removal of responsibility, in which contributors described that ‘Problems are elsewhere’. The introduction of the electronic Medicines Book for pigs – a way of recording ABU on UK commercial and smallholder pig farms (AHDB Pork, Undated) – was described by contributors along both ends of this spectrum. At one end – furthest toward the current theme – ABU recording was encouraged not to stimulate changes in ABU practices, but to alleviate a perception amongst the general public that those keeping pigs may be responsible for ABR-related problems:

*“The new Electronic Medicine Book (eMB), that will allow us to counter criticism that British pigs are part of the problem of developing antimicrobial resistance.”* (Smallholder Writer, Practical Pigs, Spring 2017)

That being said, the theme ‘Antibiotic stewardship’ appeared alongside ‘Problems are elsewhere’ in 24 of 28 articles, showing that some reference to appropriate ABU practices could be identified in the majority of cases. Further, it is possible that the contributor in the quote above was attempting to provide an alternative motivator for recording ABU. We identified three sub-themes from this theme. Each sub-theme differed in the societal group from which blame was being lessened in terms of antibiotic overuse and ABR.

#### Sub-theme 1: British farming is not to blame

The first sub-theme involved descriptions that British farming, as a whole, should not be held responsible for human ABR. This sub-theme was often articulated by smallholder writers, journalists or farming bodies also serving commercial farmers. Here, contributors commended the actions the farming sector as a whole had already taken to reduce ABU which they described as, primarily, being undertaken to maintain consumer confidence rather than to impact ABR [see also (4))]. In the following quote, which shows elements from all three themes, the contributor assures readers of ‘Antibiotic stewardship’ measures already in place, in the context of the perceived importance of antibiotics for farm animal health and outlines their views around a lack of evidence that livestock ABU impacts human ABR.

*“In order to reduce suffering and disease and thus enhance welfare, antibiotics are used after a veterinary diagnosis and when necessary in farm animals - there is no science to support the opinion that antibiotic use in food animals creates resistance in humans. Therefore, when a farm animal gets an infection it may be diagnosed by a vet and prescribed an antibiotic; when better, the animal undergoes a withdrawal time to ensure that the antibiotic has (a) done its work and (b) no residues remain to enter the food chain."* (Veterinarian, Country Smallholding, August 2015)

This sub-theme was similar to Morris et al.’s (32) ‘Maintaining the status quo’ frame, which undermined scientific evidence linking intensive agriculture to human disease, as well as their ‘Voluntary action’ frame which downplayed the nature and scale of the risk that livestock ABU may pose to humans whilst offering alternative motivations for action. Similar themes describing scepticism expressed by UK veterinarians and farmers about the threat posed by agricultural ABU – especially in relation to human health – have also been reported by several others (7, 10, 61, 71).

#### Sub-theme 2: smallholding is not to blame

Within Sub-theme 2, contributors described that smallholders could not bear responsibility for the antibiotic overuse prevalent within commercial farming, after decades of antibiotic reliance and intensification in the commercial sector. For example, despite ABU for growth promotion being illegal in the UK since 2006 (78), a view by contributors that such a practice was prolific in commercial farming was apparent:

*“For many years, antibiotics have been used as growth promoters in commercially-produced chickens, to improve growth rates, gut health and ultimately profits.”* (Journalist, Practical Poultry, Spring 2016)

Contributors most frequently expressing this sub-theme were smallholder writers, medical professionals and those in academia. The sub-theme was commonly accompanied by the sentiment that the overuse of antibiotics by the commercial farming sector represented a significant cause of human ABR and, reflective of findings in a related work (12), within this sub-theme, contributors characterised ABU on smallholdings as fundamentally different to ABU on commercial farms. This was due to exhibiting different ABU practices and by virtue of operating different livestock-keeping systems.

“*As smallholders, we like to think we are responsible in our antibiotic use because the range we use is quite limited and also because we are usually only attending to just one or two poorly animals at a time and therefore only giving medicines to those one or two. Smallholders also don’t tend to use antibiotics to medicate against things that ‘might’ happen, whereas in commercial farming this is common.”* (Smallholder Writer, Country Smallholding, September 2017)

Possibly reflective of the complex relationships between smallholders and commercial farmers described by Holloway (23), this contrasted distinctly with the sentiment expressed in Theme 1 (‘Antibiotic stewardship’) – that smallholders and commercial farmers should consider themselves ‘in the same boat’ in terms of ABU- and ABR-related issues.

This sub-theme showed distinct similarities to the ‘system failure’ frame described by Morris et al. (32) in which “intensive farming has failed as a system of agriculture […] as it requires prolific ABU to control and prevent disease which has exacerbated the development of antibiotic-resistant bacteria and its attendant problems for human health” (p. 47). The current sub-theme could be considered a progression of the ‘system failure’ frame but through a smallholding lens, seeking to validate those attempting to provide meat through less commercial means which are not perceived to be contributing to ABR.

#### Sub-theme 3: I am not to blame

In the final sub-theme, contributors – in this case smallholders – described a sub-group of ‘other’ smallholders whom they perceived to use antibiotics inappropriately, in ways distinct from their own practices. This sub-theme appeared consistently alongside ‘Antibiotic stewardship’, as contributors encouraged these ‘other’ smallholders to change their ABU practices. Commonly, contributors described ‘other’ smallholders as exhibiting antibiotic overuse not under the supervision of a veterinarian, reflective of some of the ‘Instances of ABU’ described under Theme 2, Sub-theme 2.

*“I wince when I venture onto social media… Novice pig keeper who prefers to turn to the internet rather than go to the expense of consulting a vet. Then… the advice comes flooding in: ‘Give it some antibiotics – just to be sure. It won’t do any harm.’”* (Smallholder Writer, Country Smallholding, Spring 2017)

This sub-theme was also reflective of findings from Holloway (23) who described how smallholding participants in their study discussed a similar subset of smallholders – ‘other’ from the participants themselves – who were poorly informed about biosecurity issues and were therefore likely to be non-compliant with biosecurity best practices (23). Similar themes have also been identified by those researching UK farming ABU more generally. Coyne et al. (71) discussed how participating pig farmers regarded a minority subset of farmers as using antibiotics to compensate for poor management. Doidge et al. (30) described how sheep farmers participating in their study distanced themselves from those farmers they thought overused antibiotics on poorly managed farms.

#### Theme 3 motivations

Although not explicitly described by contributors, attribution of blame to others for the problem of overuse of antibiotics and resultant ABR may demonstrate a motivation for inaction amongst contributors in terms of improving antibiotic stewardship. For example, the appearance of Theme 3, Sub-theme 1 (‘British farming is not to blame’) alongside Theme 2, Sub-theme 3 (‘The general necessity of antibiotics for farm animal health’) – where animal welfare could be harmed if ABU reductions were not completed carefully – appeared to demonstrate a reason for lack of action to improve antibiotic stewardship beyond the interventions which had already been completed.

Ritter et al. (79) discussed the factors necessary for behavioural change amongst farmers in the context of management-based strategies for infectious disease prevention and control. Firstly, farmers must believe that their current situation is problematic and, secondly, they must perceive that they have responsibility for the problem. So-called ‘other-blaming’, however, allows actors to reduce culpability for a problem, meaning they may feel less responsibility to change their actions. This concept has been identified within UK farming ABU research (9, 10, 58, 59, 71) as well as amongst the general public (29).

That being said, the placement of blame onto certain groups – rather than the contributor’s own – may have also acted to promote change among audiences. For example, contributors in Sub-theme 3 (‘I am not to blame’) wrote in a way that denormalised the ABU practices they considered inappropriate, such as administering antibiotics without veterinary supervision. By detailing this alongside ‘Antibiotic stewardship’ measures, contributors offered their readers an alternative path.

## Conclusions–recommendations arising from this study

By completing a qualitative content analysis examining material relating to the issues of ABU and ABR over five years of smallholding print media, we have been able to triangulate findings with related work examining ABU on smallholdings (12, 24). This has allowed us to evaluate recommendations derived from these related works in the light of the findings of our content analysis. We have also been able to suggest further recommendations for how educational material aimed at smallholders on ABU and ABR topics could be adapted to encourage antibiotic stewardship.

Firstly, we consider the recommendations arising from this study for future adaptations to educational material: when considering the issue of ‘other-blaming’ within smallholding print media, it is important to remember the complexities and evidence gaps that exist around the precise significance of farm animal ABU to human ABR (80, 81) which may have led to this and similar themes being identified through other UK farming research (9, 10, 58, 59, 71). Such evidence gaps were particularly notable a decade ago when our reading frame began. Looking forward, it is now generally accepted that ABU in farm animals should be optimised for reasons of ABR (68) and findings from the current study as well as related work suggests that antibiotic stewardship could be improved on smallholdings (12, 24).

Whilst it is important to note that promoting awareness of antibiotic stewardship techniques may not necessarily provoke action (7), an association between knowledge of ABU/ABR and lower ABU has been reported for commercial farms in the Netherlands (82). Under Theme 2, Sub-theme 2 (instances of ABU), contributors appeared to complete non-veterinary-supervised-ABU in several of the instances of ABU reported. Although other reasons for non-veterinary-supervised ABU were also identified, Scott et al. (12) found that some smallholders appeared unaware that antibiotics prescribed for particular conditions should not be used for alternative indications or on different animals without veterinary oversight. Therefore, it may be pertinent for those developing educational resources to encourage smallholders to seek veterinary supervision prior to administering antibiotics kept on-site, for alternative indications or on different animals than they have been prescribed.

Given the appearance of scientific errors which we identified within the analysed publications, those developing educational material for smallholders should also consider implementing a procedure to fact-check articles and correct errors. Material should be checked to ensure that articles do not encourage practices considered inappropriate in terms of ABU, such as using antibiotics as a routine whole-herd measure aimed at preventing future infections or using EMA Category B or C antibiotics as first-line treatments when an EMA Category D antibiotic (suitable for first-line use) would be as appropriate.

Finally, it may be pertinent for those developing educational material to consider how they could engender action amongst smallholder audiences by encouraging collective responsibility for the need for antibiotic stewardship. Examples of contributors expressing a feeling of collective responsibility were demonstrated in Theme 1 (Antibiotic stewardship), where contributors accepted a level of responsibility for human ABR, in terms of their own antibiotic stewardship or the encouragement of antibiotic stewardship among others. Such material could also involve case studies, in which smallholders describe actions they have taken to improve antibiotic stewardship or reduce inappropriate ABU.

Moving onto how our findings triangulate between related works, our study identified discrepancies between contributors’ recommendations for antibiotic stewardship – described under Theme 1 – and contributors’ accounts of ABU practices on smallholdings – demonstrated by Theme 2, Sub-theme 2. For example, under Theme 2, Sub-theme 2, ‘Instances of ABU on smallholdings’ appeared to be in response to non-specific clinical signs of disease (e.g., a high rectal temperature) in six out of 16 instances; instances of ABU appeared targeted toward a most likely diagnosis in four out of 16 cases. Without prior completion of a clinical reasoning approach to determine the most likely diagnoses and suitable antibiotic therapy, antibiotics may be used when they are not required or the most appropriate type of antibiotic may not be chosen (68). These findings are reflective of results from semi-structured interviews completed with smallholders who characterised ABU on smallholdings as a first-line approach to non-specific clinical signs of disease or to prevent future infections (12). Veterinarians have also described the commonness of ‘just in case’ ABU on smallholdings (24). These findings reinforce the need to develop evidence-based interventions to improve the completion and communication of a clinical reasoning approach to guide antibiotic-related decisions on smallholdings. As recommended in a related work (24), such a process may include supporting veterinarians to make antibiotic-related decisions through the collaborative development of clinical problem and species-specific clinical guidelines [see also (83)]. As this barrier to antibiotic stewardship appears consistent across findings in the UK commercial farming sector (10, 11, 64), it is possible that interventions being trialled for the farming sector more generally could be adapted for the UK smallholding context (5, 84).

Instances of ABU described by smallholders which did not appear to be under veterinary supervision are reflective of findings in research examining both smallholder and veterinary perspectives around ABU on smallholdings as well as research examining ABU on UK commercial farms (11, 12, 24). Therefore, findings of our study reinforce the need to develop interventions aimed at improving veterinary oversight of farm animal ABU. As suggested in a related work and by other UK farming antibiotic-related research, such interventions could include the collaborative creation of veterinary health plans by veterinarians and smallholders (12, 27). Plans could detail how antibiotics should be used on the holding and when veterinary advice should be sought.

### Limitations

Written sources have been described to potentially privilege elite voices (85), meaning that data cannot be assumed to be generalisable to an entire community (45, 86). We only examined print media, although it is likely that smallholders discuss and consult on matters of ABR and ABU via many other sources. For this and other reasons, the findings from our study may not be representative of a wider cohort of smallholders but may only represent the views of contributors to the chosen print media sources.

Clarification or further questioning of the contributors’ chosen language was not possible with our methodology (45, 86). Possibly reflecting this, some articles were difficult to assign to themes. The lack of dialogue between the researcher and participants may also have left meaning open to misinterpretation (45, 86) and other researchers may have come to different conclusions. Given that our reading frame was from 2015 to 2020, it is also possible that some of the recommendations derived from this study are no longer relevant as they may have already been enacted, given the fast-paced nature of progressions within the field of farm animal antibiotic stewardship.

That being said, whilst these and other limitations to analysing secondary sources exist, our use of a qualitative content analysis has enabled triangulation between related studies involving semi-structured interviews to a more geographically disparate group of smallholders. This method may have also allowed us to access harder-to-reach participants, who may not have wanted to partake in the interviews conducted in related studies examining ABU on smallholdings (12, 24).

## Data Availability

Publicly available datasets were analyzed in this study. These data can be found at: https://pocketmags.com/; https://shop.kelsey.co.uk/; https://www.magzter.com/.
